# Aldosterone Biosynthesis Is Potently Stimulated by Perfluoroalkyl Acids: A Link between Common Environmental Pollutants and Arterial Hypertension

**DOI:** 10.3390/ijms24119376

**Published:** 2023-05-27

**Authors:** Brasilina Caroccia, Teresa Maria Seccia, Giorgia Pallafacchina, Maria Piazza, Ilaria Caputo, Stefania Zamberlan, Rosario Rizzuto, Gian Paolo Rossi

**Affiliations:** 1 Internal Emergency Medicine Unit, Specialized Center for Blood Pressure Disorders-Regione Veneto, Department of Medicine—DIMED, University of Padua, 35131 Padua, Italymaria.piazza@unipd.it (M.P.);; 2 Department of Biomedical Sciences—DSB, University of Padua, 35131 Padua, Italy; giorgia.pallafacchina@unipd.it (G.P.);; 3 Neuroscience Institute, Italian National Research Council (CNR), 35131 Padua, Italy

**Keywords:** perfluoroalkyl substances, endocrine disruptors, aldosterone, aldosterone synthase gene expression, arterial hypertension, reactive oxygen species

## Abstract

The large environmental contamination of drinking water by perfluoroalkyl substances (PFAS) markedly increased the plasma levels of pentadecafluorooctanoic acid (PFOA) and perfluorooctanesulfonic acid (PFOS) in a Northern Italy population with a high prevalence of arterial hypertension and cardiovascular disease. As the link between PFAS and arterial hypertension is unknown, we investigated if they enhance the biosynthesis of the well-known pressor hormone aldosterone. We found that PFAS increased aldosterone synthase (*CYP11B2*) gene expression by three-fold and doubled aldosterone secretion and cell and mitochondria reactive oxygen species (ROS) production over controls (*p* < 0.01 for all) in human adrenocortical carcinoma cells HAC15. They also enhanced the effects of Ang II on *CYP11B2* mRNA and aldosterone secretion (*p* < 0.01 for all). Moreover, when added 1 h before, the ROS scavenger tempol abolished the effect of PFAS on *CYP11B2* gene expression. These results indicate that at concentrations mimicking those found in human plasma of exposed individuals, PFAS are potent disruptors of human adrenocortical cell function, and might act as causative factors of human arterial hypertension via increased aldosterone production.

## 1. Introduction

Natural or man-made chemicals acting as receptor ligands, and/or affecting the synthesis, plasma levels, metabolism and/or clearance of hormones are defined as endocrine-disruptor compounds (EDCs) [1,2]. Humans are widely exposed to the action of EDCs because EDCs are extensively present in food and contact materials, cosmetics, household goods, and toys [1]. Epidemiological evidence of an association between EDC exposure and diminished quality of life, lower cancer-free survival, cardiovascular and endocrine diseases [3], including obesity, type 2 diabetes mellitus, and the metabolic syndrome exist [1,2,4,5].

Based on evidence linking perfluoroalkyl substances (PFAS) with adverse effects on general health and endocrine system, the World Health Organization included them among the EDCs [1,2]. Due to their chemical and thermal stability, along with water- and oil-repellence properties, PFAS are widely used in industrial processes and commercial products [6,7], which resulted in their wide dispersion in the biosphere and the contamination of living organisms, such as fish, birds, mammals, and humans, by the ingestion of contaminated water or food [8,9]. This is a matter of much concern because, once ingested, PFAS are poorly metabolized and excreted and persist at detectable levels for years in blood, tissues and organs, with an estimated elimination time from human plasma ranging from 5 to 8.5 years [10].

As in *Erin Brockovich*, the movie directed by Steven Soderbergh in 2000, an industrial contamination with ensuing environmental pollution by PFAS has become a major public health issue in the Veneto Region of North-Eastern Italy, because over a million residents were exposed to PFAS through drinking water for decades, until autumn 2013, when an EU initiative discovered and stopped the pollution. At that time, systematic measurements of circulating PFAS levels in all inhabitants of the contaminated municipalities were used to identify that the PFAS with the highest serum concentrations were pentadecafluorooctanoic acid (PFOA) and perfluorooctanesulfonic acid (PFOS) [11,12]. Of note, the PFOA and PFOS blood concentrations were, respectively, 8 and 1.5 times higher in residents of contaminated areas compared to that found in unexposed populations [11]. Epidemiological data collected during the period 1980–2013 showed that inhabitants of the polluted area had higher relative risks of death, diabetes mellitus, cerebrovascular diseases, myocardial infarction, Alzheimer’s and Parkinson’s diseases, kidney, and breast cancer, than residents exposed to similar socio-economic conditions and smoking habits living in nearby municipalities but using uncontaminated water [13].

A health surveillance program, established to aid in the prevention, early diagnosis and treatment of chronic disorders possibly associated with PFAS exposure [12], demonstrated that serum PFOA and PFOS levels were directly related to increased systolic and diastolic blood pressure (BP) values and the prevalence of arterial hypertension [14]. However, whether these associations were just chance findings or had a mechanistic explanation remained unknown.

PFAS were prevoiusly reported to affect estradiol and testosterone release by Du et al. [15] in H295 cells, an adrenocortical carcinoma cell line, where PFOA, at a concentration ranging from 3 nM to 300 nM, also altered the expression of major steroidogenic genes (*StAR*, *17bHSD1*, *CYP19*, *3bHSD2*), and of the steroidogenic factor SF-1. In 2016, Kang et al. [16] investigated the effects of PFOA and PFOS on the synthesis of sex hormones in H295R cells, and incidentally they reported that PFAS increased *CYP11B2* and *CYP11B1* mRNA, and confirmed their effect on *CYP19* and *3bHSD2*, as previously reported by Du et al. [15]. These data clearly suggest that PFAS could modulate steroidogenesis and act as potent endocrine disruptors in the adrenal gland. However, there is no information on PFAS’ effect on aldosterone release and blood pressure, and no data on the mechanisms by which PFAS can induce aldosterone biosynthesis exist.

We hypothesized that PFAS enhance aldosterone biosynthesis, and thereby raise blood pressure. Since aldosterone production occurs in mitochondria and requires a highly oxidative environment [17], and PFAS were described to promote oxidative stress [18], we wondered whether PFAS exposure could increase the generation of reactive oxidative species in the mitochondria of human adrenocortical cells. To verify this hypothesis, we investigated the effect of the two main PFAS pollutants on aldosterone synthase gene expression, aldosterone secretion, and mitochondrial oxidative stress in human adrenocortical cells, HAC15, a clone derived from H295R cells that produces more aldosterone in vitro and shows the best ability to respond to Ang II when compared with the other available human adrenocortical cell lines [19]. Furthermore, considering that angiotensin (Ang) II stimulates aldosterone biosynthesis under both physiological and pathophysiological conditions, we also evaluated whether PFAS potentiate the effect of this aldosterone secretagogue.

## 2. Results

### 2.1. PFAS Do Not Affect the Cell Viability of Adrenocortical Cells

To evaluate if PFOA and PFOS, alone or in combination, affected cell viability, HAC15 cells were treated with 1 μM or 10 μM PFOA, PFOS or both, and compared to vehicle-treated cells. The methyl-thiazol-etetrazolium (MTT) colorimetric assay was performed at 24, 48, or 72 h, and showed >95% cell viability at all times and in all conditions, thus ruling out any effects of PFOA and PFOS, either alone or in combination, on HAC15 cell viability (Supplemental Figure S1a–c).

### 2.2. PFAS Enhance Aldosterone Synthase Gene Expression

**Figure 1:**
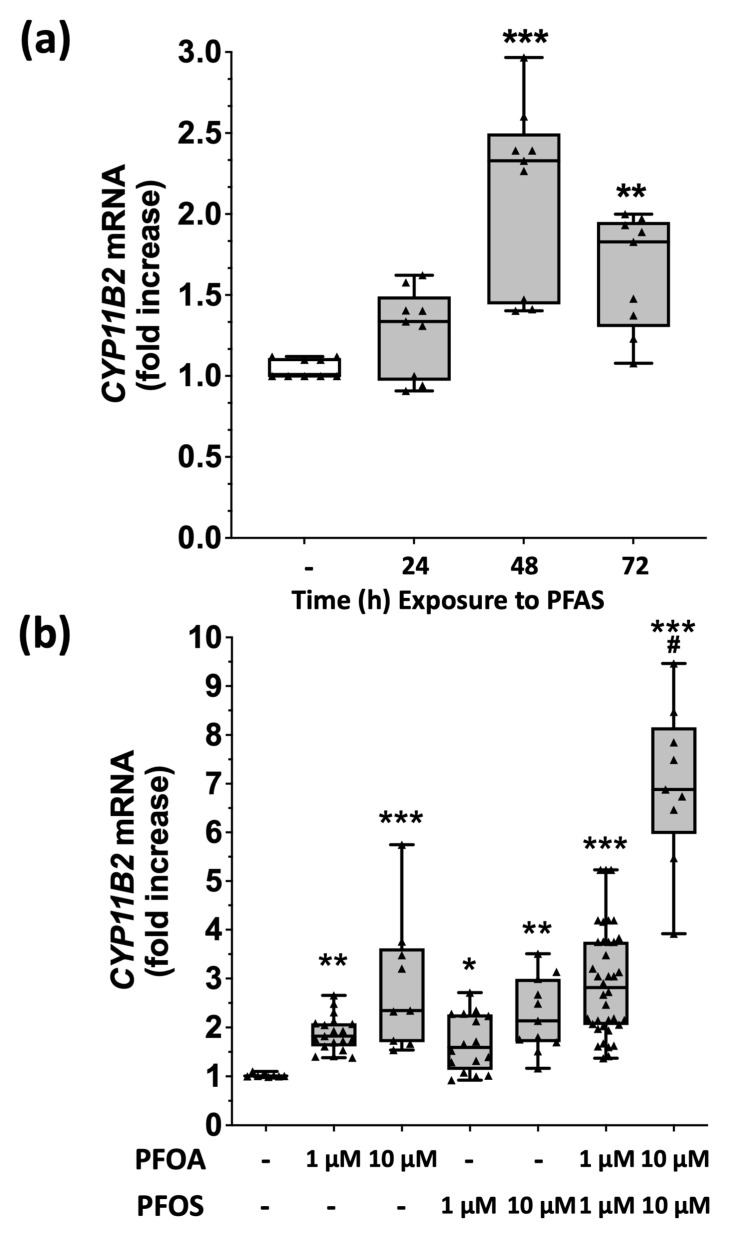
PFAS enhances aldosterone synthase gene expression in a concentration-dependent manner. Panel (**a**): Aldosterone synthase (CYP11B2) gene expression, evaluated by real-time PCR, was increased in HAC15 cells after treatment with 1 μM PFOA + 1 μM PFOS, with a peak effect at 48 h. All data represent median and 95% CI of at least 3 experiments, each performed in triplicate. ** *p* < 0.01 vs. vehicle; *** *p* = 0.001 vs. vehicle. Panel (**b**): Using two different concentrations (1 μM and 10 μM) of PFOA or PFOS or a combination of the two compounds, CYP11B2 gene expression was found to be enhanced in a dose-dependent manner at 48 h. * *p* < 0.05 vs. vehicle; ** *p* < 0.01 vs. vehicle; *** *p* < 0.001 vs. vehicle; # *p* < 0.001 vs. PFAS 1 μM.

We then assessed the effect of 48 and 72 h of treatment with 1 μM PFOA + PFOS on aldosterone synthase (CYP11B2) mRNA levels. By using real-time PCR, we found that PFAS significantly enhanced CYP11B2 gene expression (Figure 1a), with a peak effect at 48 h. To clarify if the effect on CYP11B2 gene expression was determined by PFOS or PFOA, we next treated HAC15 cells with each PFAS, alone or together, at 1 and 10 μM concentrations for 48 h, and found that both PFOA and PFOS increased CYP11B2 gene expression in a concentration-dependent manner (Figure 1b). Moreover, the combined treatment with PFOA + PFOS at both 1 μM and 10 μM enhanced CYP11B2 gene expression over each PFAS alone (Figure 1b).

### 2.3. PFAS-Induced Aldosterone Biosynthesis Involves Enhanced Whole-Cell and Mitochondrial Reactive Oxygen Species Production

**Figure 2:**
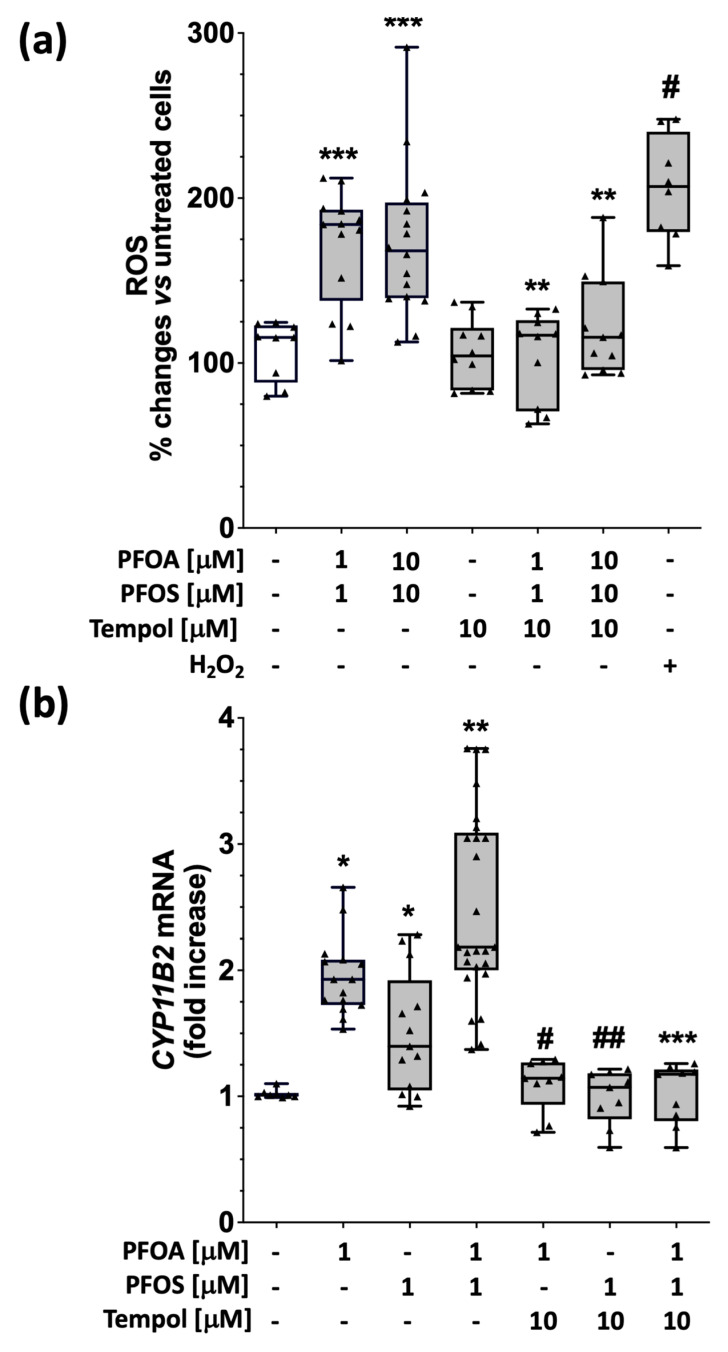
PFAS enhances ROS production and aldosterone synthase gene expression in HAC15 cells through a mechanism blunted by the ROS scvanger tempol. Panel (**a**): Percent changes in ROS production increased after 48 h treatment with 1 μM or 10 μM of PFAS (PFOA + PFOS) or H_2_O_2_ (used as positive control) compared to vehicle (*** *p* < 0.001, # *p* < 0.001), while 10 μM tempol abolished this effect (** *p* < 0.01 vs. PFAS). Panel (**b**): Fold increase from vehicle-treated cells of CYP11B2 mRNA after treatment with PFOA, PFOS and PFOA + PFOS (1 μM or 10 μM) (* *p* < 0.05 vs. vehicle, ** *p* < 0.01 vs. 1 μM PFOA and vs. 1 μM PFOS); the pre-treatment with 10 μM tempol abolished the effect of PFOA, PFOS and PFOA + PFOS (1 μM or 10 μM). Data represent median and 95% CI of at least 3 experiments performed in parallel in the same cell batch in triplicate; # *p* < 0.001 vs. 1 μM PFOA, ## *p* < 0.01 vs. 1 μM PFOS, *** *p* < 0.001 vs. 1 μM PFOA + PFOS.

We measured whole-cell ROS levels by dihydroethidium (DHE) fluorescent probe in HAC15 cells treated with 1 μM and 10 μM PFOA + PFOS for 48 h to investigate if PFOA and PFOS enhanced reactive oxygen species (ROS) generation, as the final step of aldosterone synthesis is an O^2−^-dependent reaction [17]. Not only did we find that 1 μM and 10 μM PFOA + PFOS increased intracellular ROS, we also found that this increase was abolished by the superoxide scavenger Tempol (Figure 2a) that was added to cell medium 1 h before PFAS.

To confirm that PFOA- and PFOS-induced ROS generation was responsible for the enhanced CYP11B2 gene expression, we measured CYP11B2 transcript levels in HAC15 cells treated with 1 μM PFOA and/or 1 μM PFOS in the presence of 10 μM Tempol. The results showed that 10 μM Tempol abolished the effect of PFOA, PFOS and PFOA + PFOS on CYP11B2 gene expression (Figure 2b), indicating that PFAS-dependent ROS production is necessary for CYP11B2 transcription.

**Figure 3:**
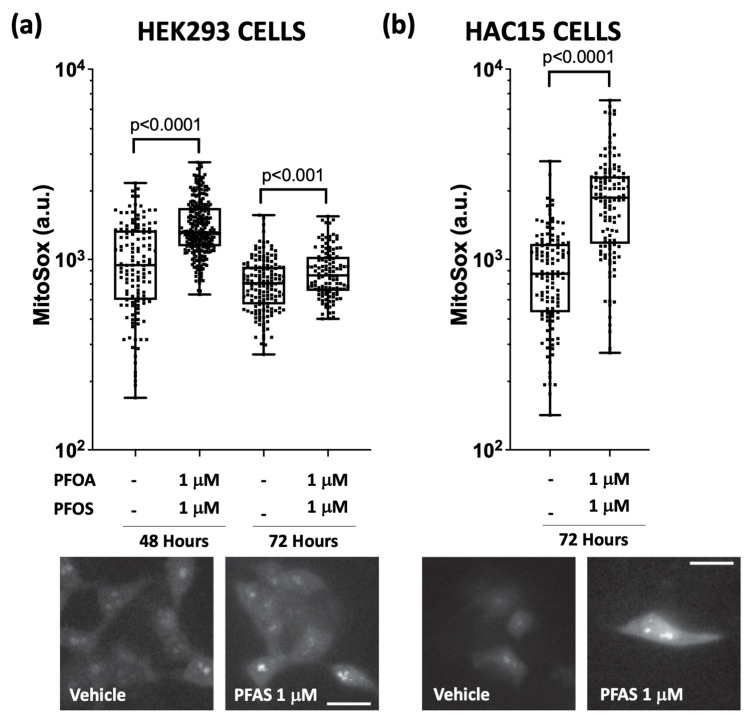
PFAS enhances ROS formation in the mitochondria. Panel (**a**): ROS formation in the mitochondria was measured with MitoSox, a mitochondria-specific probe, in HEK293 cells. Mitochondrial ROS production was higher in cells treated with 1 μM PFOA + PFOS for 48 and 72 h compared to vehicle. Microphotographs are representative of HEK293 cells treated with vehicle or 1 μM PFOA + PFOS and loaded with MitoSox probe. Three different experiments were performed and at least eight different fields per coverslip were analysed (median and 95% CI). Panel (**b**): Mitochondrial ROS production was increased in HAC15 cells treated with 1 μM PFOA + PFOS for 72 h. Microphotographs show HAC15 cells treated with vehicle or 1 μM PFOA + PFOS and loaded with MitoSox probe. Three different experiments were performed and at least eight different fields per coverslip were analysed (median and 95% CI).

As the final steps of aldosterone biosynthesis occur in the mitochondria [17], we performed a proof-of-concept experiment by measuring mitochondrial ROS production after PFAS exposure in HEK293 cells, which are a widely used model to investigate mitochondrial biology and mitochondrial ROS production [20,21]. HEK293 cells treated with 1 μM PFOA + PFOS for 48 and 72 h were labelled with dye (MitoSOX™ Red fluorogenic) that selectively targets mitochondria and reveals the superoxide. The results showed that mitochondrial ROS levels were significantly higher in PFAS-treated cells than in mock-treated cells (Figure 3a).

These findings were further confirmed in HAC15 cells, which showed increased mitochondrial ROS production after treatment with 1 μM PFOA + PFOS for 72 h. Thus, PFAS effect on human cells, including adrenocortical cells, involves an enhanced mitochondrial ROS production (Figure 3b).

### 2.4. PFAS Strengthen the Ang II-Induced Aldosterone Production

Ang II is one of the most studied of the multiple secretagogues that regulate the biosynthesis of aldosterone under physiological and pathophysiological conditions. It acts via the AT1R and increases superoxide generation, which is needed to enhance aldosterone synthesis [22,23,24]. Therefore, we further investigated if PFOA and PFOS could affect Ang II-induced CYP11B2 gene expression and aldosterone secretion.

**Figure 4:**
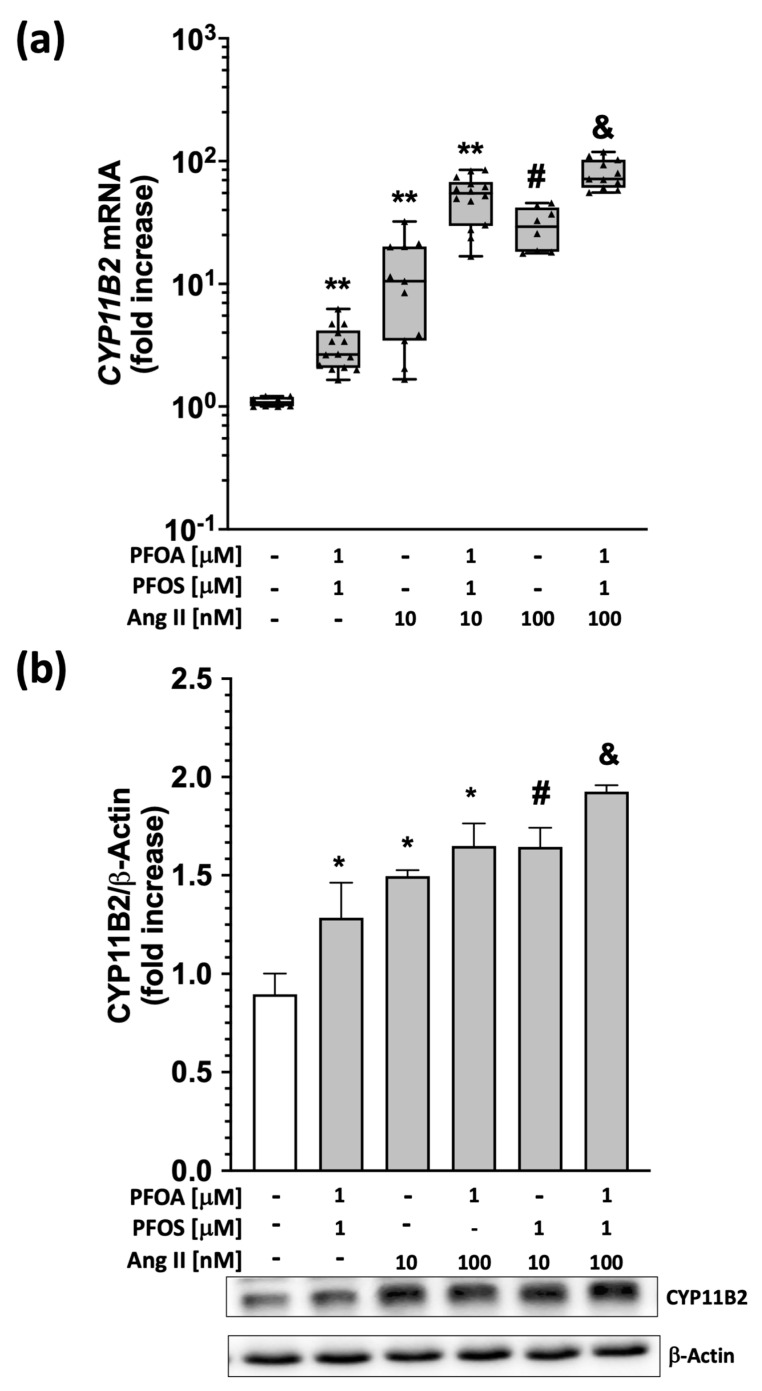
PFAS strengthen the effects of Ang II on aldosterone synthase mRNA and protein levels. Aldosterone synthase (CYP11B2) gene (Panel (**a**)) and protein (Panel (**b**)) expression was increased in HAC15 cells treated with two different concentrations of Ang II (10 nM and 100 nM), alone or on top 1 μM PFOA +PFOS (48 h of pretreatment). Panel (**a**): ** *p* < 0.01 vs. vehicle, # *p* < 0.01 vs. Ang II 10 nM, & *p* < 0.01 vs. Ang II 100 nM); data represent median and 95% CI of at least 4 different experiments, each performed in triplicate. Panel (**b**): * *p* < 0.05 vs. vehicle, # *p* < 0.05 vs. Ang II 10 nM, & *p* < 0.05 vs. Ang II 100 nM); data represent media ± SD of 3 different experiments, each performed in duplicate.

To achieve this aim, HAC15 cells were treated for 48 h with 1 μM PFOA + 1 μM PFOS, after which 10 nM or 100 nM Ang II was added for 12 or 24 h to evaluate CYP11B2 transcript levels and aldosterone synthase protein levels and aldosterone secretion, respectively. The results showed that 10 nM or 100 nM Ang II increased CYP11B2 mRNA by 12- and 52-fold, respectively, compared to vehicle (Figure 4a). Of note, 1 μM PFOA + 1 μM PFOS potentiated the effect of 10 nM or 100 nM Ang II on CYP11B2 gene expression by 30- and 80-fold, respectively (Figure 4a). These results were confirmed at the protein levels, as shown in Figure 4b. Aldosterone synthase protein expression was significantly enhanced by PFOA and PFOS (1 μM each) and Ang II (10 or 100 nM). Moreover, Ang II’s effect was strengthened by PFOA and PFOS (1 μM each).

**Figure 5:**
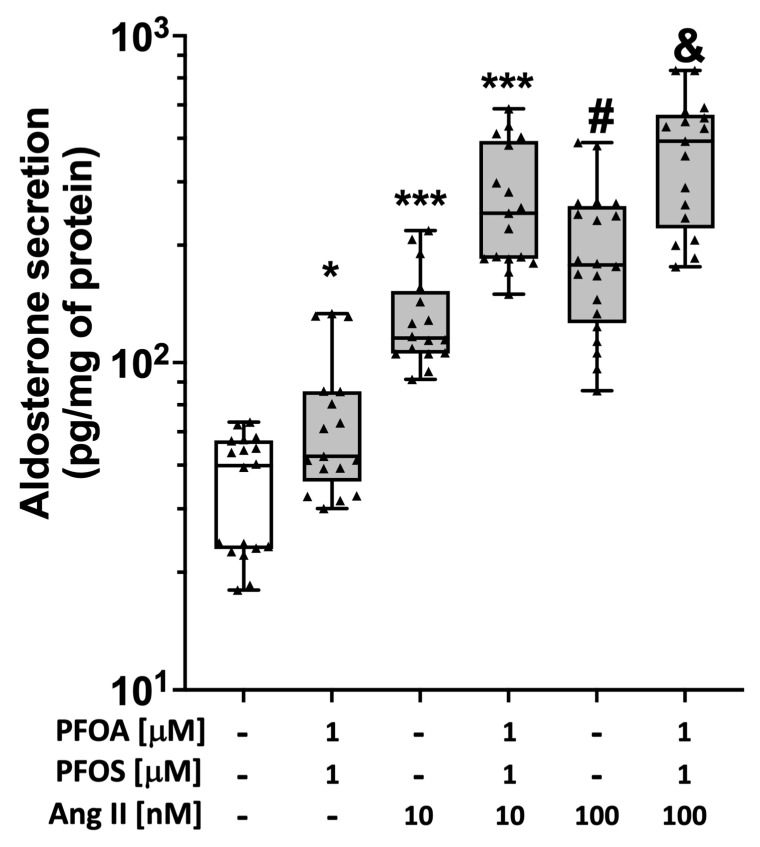
PFAS strengthen the effects of Ang II on aldosterone secretion. Aldosterone levels were increased in HAC15 cells treated with two different concentrations of Ang II (10 nM and 100 nM) alone or after 48 h of treatment with 1 μM PFOA + PFOS. Data represent median and 95% CI of at least 4 different experiments, each performed in triplicate. * *p* < 0.05 vs. vehicle, *** *p* < 0.001 vs. vehicle, # *p* < 0.05 vs. Ang II 10 nM, & *p* < 0.05 vs. Ang II 100 nM).

Combined treatment with PFOA and PFOS (1 μM each), or 10 nM or 100 nM Ang II significantly increased aldosterone secretion compared to vehicle (Figure 5). This effect was stimulated more markedly by the combination of PFOA + PFOS (1 μM each) and Ang II than by Ang II alone (either 10 nM or 100 nM) (Figure 5).

**Figure 6:**
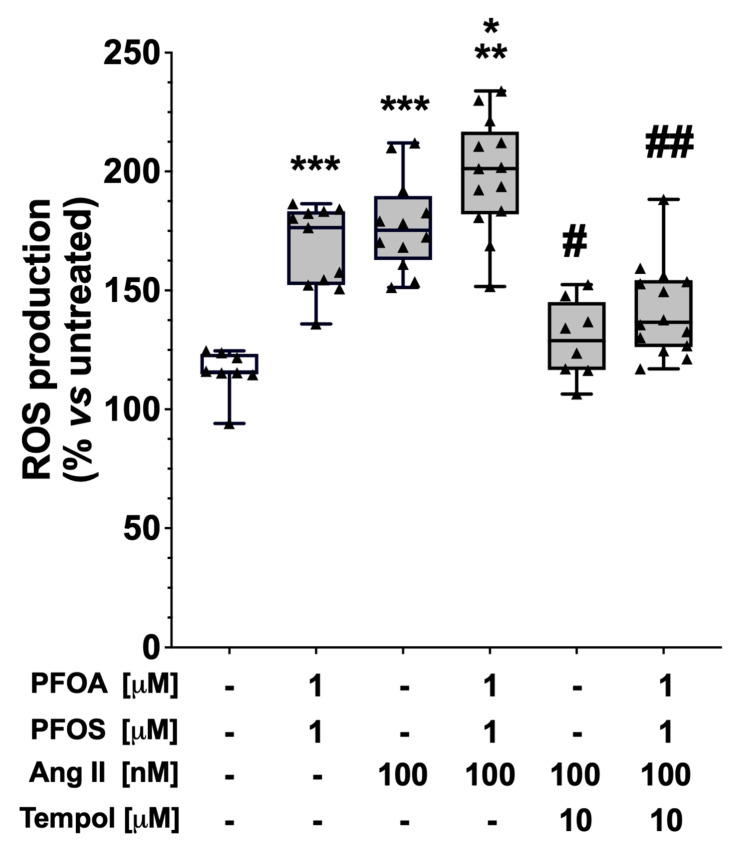
PFOA, PFOS and Ang II increased ROS production in HAC15 cells after 48 h. Ang II 100 nM was added for 6 h on top of 1 μM PFOA + PFOS and ROS production was increased compared to 1 μM PFOA + PFOS and Ang II alone. The increase in ROS induced by Ang II + 1 μM PFOA + PFOS was blunted by ROS scavenger tempol. Data are presented as percentage changes from vehicle-treated cells (median and 95% CI of at least 3 experiments, each performed in triplicate). *** *p* < 0.001 vs. vehicle-treated cells; ** *p* < 0.01 vs. 1 μM PFOA + PFOS; * *p* = 0.03 vs. Ang II; # *p* < 0.001 vs. Ang II; ## *p* < 0.001 vs. Ang II + 1 μM PFOA + PFOS.

To clarify whether PFAS potentiated the effect of Ang II by increasing ROS production, we measured whole-cell ROS levels by DHE probe in HAC15 cells treated with 1 μM and 10 μM PFOA + PFOS for 48 h, with and without the addition of Ang II, in the presence and absence of Tempol. We found that 100 nM Ang II significantly increased intracellular ROS, which were effectively scavenged by Tempol (Figure 6). Pre-treatment with 1 μM PFOA + PFOS slightly augmented Ang-II-induced ROS production while Tempol abolished it (Figure 6), thus confirming that both Ang II and PFAS act via generation of ROS.

## 3. Discussion

Considering the widespread use of PFAS in food and contact materials, cosmetics, household goods, and toys, humans are widely exposed to the action of these substances [1]. Our study showed, for the first time, that the two most prevalent PFAS detected in the plasma of a large population exposed to long-standing environmental pollution enhance aldosterone secretion via ROS production and thus act as potent endocrine disruptors. At concentrations chosen to mimic those found in exposed workers of the polluted ‘red zone’, which are similar to those found in the contaminated area of West Virginia and Ohio [25], both PFOA and PFOS markedly increased CYP11B2 gene expression and aldosterone release in human adrenocortical HAC15 cells (Figure 1, Figure 2, Figure 4 and Figure 5). Whether a lower PFAS concentration, such as that found in the general population of the polluted area, is associated with increased plasma aldosterone levels and the development of primary aldosteronism, and whether a threshold effect exists could not be determined at this stage because plasma aldosterone and renin of the exposed population were not measured in previous studies [14].

The present findings highlight a potential mechanism whereby arterial hypertension was increased in communities exposed to these levels of environmental pollution, and suggest that they are important not just for the exposed population, but in the population at large, as they might help to explain the apparent epidemics of primary aldosteronism [26,27].

Our results also showed that PFOA and PFOS augment aldosterone biosynthesis (Figure 5) by increasing ROS production (Figure 2), as the ROS scavenger tempol restored their baseline levels, and abolished the secretagogue effect of PFAS on aldosterone (Figure 2). As the conversion of 11-deoxycorticosterone to aldosterone involves exposure to the highly oxidative environment present in the mitochondria [17], we extended our investigation to the ROS levels induced by PFAS in these organelles and found that PFAS markedly increased ROS generation in the mitochondria (Figure 3), indicating that PFAS act by increasing oxidative stress in these organelles.

Since the regulation of aldosterone biosynthesis is a highly complex process involving peptide-activated G-protein-coupled receptors and various peptide ligands that act in a concerted way [22,23], establishing whether PFAS modulate the action of known physiological stimuli of aldosterone secretion is a further important piece of knowledge. Angiotensin II, one of the best-characterized aldosterone secretagogues, acts via the angiotensin type I receptor (AT1R) and ROS generation. We found that both PFOA and PFOS, alone or in combination, markedly potentiated the secretagogue effect of Ang II on aldosterone production (Figure 5). The mechanisms underlying this potentiation, and whether PFAS could enhance the regulation of aldosterone by peptides other than Ang II, such as endothelin-1 [22,28], are important questions that deserve further ad hoc research.

The finding that PFAS directly increase aldosterone biosynthesis and augment the effect of Ang II on aldosterone release should not be disregarded as small increases in the plasma concentrations of aldosterone within the physiologic range were shown to raise blood pressure in the general population of the Framingham Offspring study [29] and to be associated with the two main detrimental consequences of high blood pressure, i.e., left ventricular hypertrophy [30] and incident atrial fibrillation in the ARIC study [31].

Since PFOA and PFOS have a very long half-life in plasma and tissues, and the AT1R is expressed in aldosterone-producing adenoma, causing primary aldosteronism [32], our results collectively suggest that PFAS could act as causative and/or enhancing factors in human hypertension due to relative or absolute aldosterone excess. These observations can be important since, although aldosteronism is quite common, little is known about its etiological/predisposing factors.

It is worth mentioning that, even though our results were obtained in vitro, their relevance is supported by our recent observation that a patient who presented with an aldosterone-producing adenoma and was cured, both biochemically and hemodynamically, with adrenalectomy showing high PFAS plasma levels. Clinical follow-up is ongoing to determine if its primary aldosteronism will recur, as the plasma and tissues PFAS levels will take years to normalize. Whether the prevalence of primary aldosteronism and aldosterone-producing adenoma is increased in the polluted red zone of the Veneto region is unknown and quite difficult to ascertain, because, primary aldosteronism is markedly underdiagnosed since it often presents itself as essential hypertension [33].

The pressor effect of PFAS is not only in line with the Veneto region data [14], but also with the observation of a significant direct association between PFAS plasma levels and blood pressure values in large, population-based, cross-sectional observational studies performed in the US [34] and China [35], and with the results of a recent 18-year prospective study in midlife women initially free of hypertension [36]. Nonetheless, as some longitudinal studies failed to confirm an association between PFAS levels and blood pressure values or the prevalence of hypertension [37,38,39], the effect of PFAS on blood pressure and aldosterone likely varies across populations, ethnicities, and salt intake, and thus underlies the multifactorial nature of arterial hypertension.

In summary, we discovered that two most widely present environmental pollutants, perfluoroalkyl substances (PFAS) pentadecafluorooctanoic acid (PFOA) and perfluorooctanesulfonic acid (PFOS), act as potent inducers of mitochondrial oxidative stress and aldosterone secretion in human adrenocortical cells. They might act as etiological and/or facilitating factors in hyperaldosteronism, both in the presence (i.e., secondary aldosteronism) and the absence of overt activation of the renin–angiotensin system (i.e., primary aldosteronism), since PFAS markedly potentiate the action of Ang II.

Of note, based on the association between PFAS exposure and adverse outcomes, some companies making PFAS voluntarily set out to replace long-chain PFAS with short-chain PFAS in recent years [40]. However, data on the biological activity of the short-chain PFAS and their long-term effects on human health are scant, thus warranting further research.

## 4. Materials and Methods

### 4.1. Cell Cultures

HAC15s were grown in DMEM/F12 (Sigma-Aldrich, Milan, Italy) supplemented with 10% cosmic calf serum (CCS) (Hyclone, Thermo Scientific, Waltham, MA, USA), 1% glutamine (Sigma-Aldrich, Milan, Italy), and 1% antibiotic/antimycotic mixture (Sigma-Aldrich, Milan, Italy). For the experiments, the cells were seeded in 12-well plates at 3 × 10^5^ cells per well and grown to sub-confluence (80%).

Human embryonic kidney 293 (HEK293) cells were grown in DMEM/F-12, GlutaMAX supplement (Gibco, Milan, Italy) supplemented with 10% FBS (Euroclone, Milan, Italy), 1% penicillin—streptomycin (Sigma-Aldrich, Milan, Italy), with fresh culture medium replenished every 2–3 days.

Prior to experiments, HAC15 cells were synchronized by incubation for 16 h with DMEM/F12 medium supplemented with 0.5% CCS or FBS, 1% glutamine and 1% antibiotic/antimycotic. They were then treated with 1 μM or 10 μM pentadecafluorooctanoic acid (PFOA, diluted in sterile water, Sigma-Aldrich, Milan, Italy) and 1 μM or 10 μM perfluorooctanesulfonic acid (PFOS, prepared in methanol, Sigma-Aldrich, Milan, Italy). Parallel experiments were conducted by exposing HAC15 cells to both substances. The concentrations of PFOA and PFOS used in our experiments were chosen based on the serum concentration found in exposed and highly exposed workers in Veneto Region [41,42].

HAC15 cells were used to investigate the effects of PFAS on *CYP11B2* gene and protein expression, aldosterone secretion, and mitochondrial ROS production. HEK293 cells were used to investigate the effect of PFOA and PFOS on mitochondrial ROS production.

### 4.2. Cell Viability Assay

Cell viability was measured with the methyl–thiazol–etetrazolium (MTT) colorimetric assay system (CalBiochem), following the manufacturer’s protocol. In brief, 5 × 10^5^ HAC15 cells were exposed for 24, 48 and 72 h to 1 μM or 10 μM PFOA, 1 μM or 10 μM PFOS and 1 μM or 10 μM PFOA + PFOS in 96-well plates. After treatment, 25 μL of the MTT solution was added to each well and incubated for 4 h at 37 °C. At the end of the incubation, the absorbance was determined at 570 nm.

### 4.3. RNA Extraction and Quantitative Real-Time PCR

RNA was extracted from cells with the Roche RNeasy kit (Roche, Monza, Italy) according to the manufacturer’s protocol. A total of 1 μg total RNA from cells was reverse-transcribed in a final volume of 20 μL using the iScript^TM^ cDNA Synthesis kit (Bio-Rad, Milan, Italy). The RT-PCR reactions were performed in Delphi 1000 TM Thermal Cycler (Oracle Biosystems^TM^, Watertown, MA, USA).

Aldosterone synthase (*CYP11B2*, NM_000498.3, primer For: 5′-gtgaccgcaggttgcttt-3′, primer Rev: 5′-cccttattcctttcccatgc-3′) mRNA levels were measured with a real-time RT-PCR by the comparative Ct (2^−ΔΔCt^) method; porphobilinogen deaminase (*PBGD*, NM_000190.3, primer For: 5′-tgccctggagaagaatgaag-3′, primer Rev: 5′-agatggctccgatggtga-3′) gene was used as a housekeeping gene. Each experiment was repeated at least five times in duplicate, and the results are presented as fold increase over vehicle-treated cells.

### 4.4. Reactive Oxygen Species Detection

Reactive oxygen species’ (ROS’) formation in HAC15 cells was determined using the dihydroethidium (DHE) fluorescent probe (Life Technologies, Milan, Italy). HAC15 cells (5 × 10^5^) were exposed for 48 h to the different concentrations of PFOA + PFOS (1 μM or 10 μM). Pretreatment with 10 μM Tempol was performed 1 h before adding PFOA + PFOS. Prior to treatment termination, 2.5 μM DHE was added to cell medium for 30 min at 37 °C, and then washed with PBS. Fluorescent intensity was read using EnSight Multimode Plate reader (PerkinElmer, Waltham, MA, USA) at the excitation and emission wavelengths of 480 and 590 nm, respectively.

### 4.5. Mitochondrial ROS Measurements

We measured mitochondrial superoxide production using the MitoSOX Red dye (Thermo Fisher, Milan, Italy). HAC15 and HEK293 cells were seeded on 24 mm coverslips at sub-confluence concentration, treated for 48 and 72 h with 1 μM PFOA + PFOS, and then incubated with MitoSOX Red (1.2 μM, Thermo Fisher, Milan, Italy) in 1 mL of KREBS ringer buffer (KRB) at 37 °C for 40 min in the dark. Before imaging, cells were rapidly washed three times in KRB and placed on an Olympus IX73 microscope with PlanFluor 40 X/1.3 N.A. objective for image acquisition.

Excitation was performed with a DeltaRAM V high-speed monochromator (Photon Technology International, Birmingham, NJ, USA) equipped with a 75 W xenon arc lamp. Images were captured with a high sensitivity Evolve 512 Delta EMCCD (Photometrics, Tucson, AZ, USA). The system was assembled by Crisel Instruments and is controlled by MetaFluor 7.5 (Molecular Devices, San Jose, CA, USA). Cells were thus illuminated at 488 nm and fluorescence was collected through a 605/52 band emission filter (Chroma Technology Corp, Bellows Falls, VT, USA). Acquisitions were performed at 20 ms exposure time. All the acquisition parameters were set according to MetaFluor 7.5 system and MitoSOX Red dye. At least eight different fields were collected per coverslip. Analysis was performed with the Fiji distribution of ImageJ v1.52 [43,44]. All images were background-corrected by subtracting mean pixel values of a cell-free region of interest. Data are presented as the mean intensity of the selected region of interest, representing the cell mitochondria.

### 4.6. Aldosterone Measurement

Aldosterone was measured with the Aldosterone Elisa kit (Alpha Diagnostic International, San Antonio, TX, USA) in the supernatant. HAC15 cells were treated with 1 μM PFOS + PFOA for 48 h. After 48 h, the cells were also exposed to 10 nM or 100 nM Ang II for an additional 24 h. At the end of the incubation time, medium was collected for aldosterone measurement and proteins were extracted from the cells for CYP11B2 protein quantification. To correct for differences in cell number per well, aldosterone levels were normalized to the protein content.

### 4.7. Immunoblotting

Proteins (20 µg) were separated on 10% SDS-PAGE gel and electro-transferred to nitrocellulose membranes (Hybond ECL-Amersham Biosciences, Amersham, UK). The membranes were blocked for 2 h with TBS 5% added with Albumin Bovine Serum, and then incubated overnight with mouse anti-CYP11B2 antibody (dilution 1:1000) (a kind gift of Prof. Celso Gomez-Sanchez, University of Mississippi Medical Center, Jackson, MS, USA). The specific immunosignal was visualized by a luminol-based chemiluminescen substrate (LumiGLO, KPL, Kitchener, ON, Canada). Images were processed by Molecular imager VersaDoc system (Biorad, Hercules, CA, USA). Bands for CYP11B2 were normalized to b-actin (rabbit polyclonal antibody from Cell Signaling, Danvers, MA, USA).

### 4.8. Statistics

Statistical analysis was performed with GraphPad Software™ (version 9.01 for Mac OS X, La Jolla, CA, USA). The comparison between vehicle- and PFOA- or PFAS-treated cells was performed with the non-parametric Mann–Whitney test for independent (unpaired) samples. One-way and repeated-measures ANOVA with post-hoc Scheffé test were used to investigate the effect of PFOA and PFAS on cell viability in the MTT experiment.

## Data Availability

The data generated in this study are available on reasonable request from the corresponding author.

## Supplementary Materials

The supporting information can be downloaded at: https://www.mdpi.com/article/10.3390/ijms24119376/s1.
