# Supplementary material for: Aldosterone Biosynthesis Is Potently Stimulated by Perfluoroalkyl Acids: A Link between Common Environmental Pollutants and Arterial Hypertension
Source: Int J Mol Sci. 2023 May 27;24(11):9376. doi: 10.3390/ijms24119376 (PMC10253916; doi:10.3390/ijms24119376)
Supplement: Supplementary file 1 [file ijms-24-09376-s001.zip › ijms-2411546-supplementary.pdf]

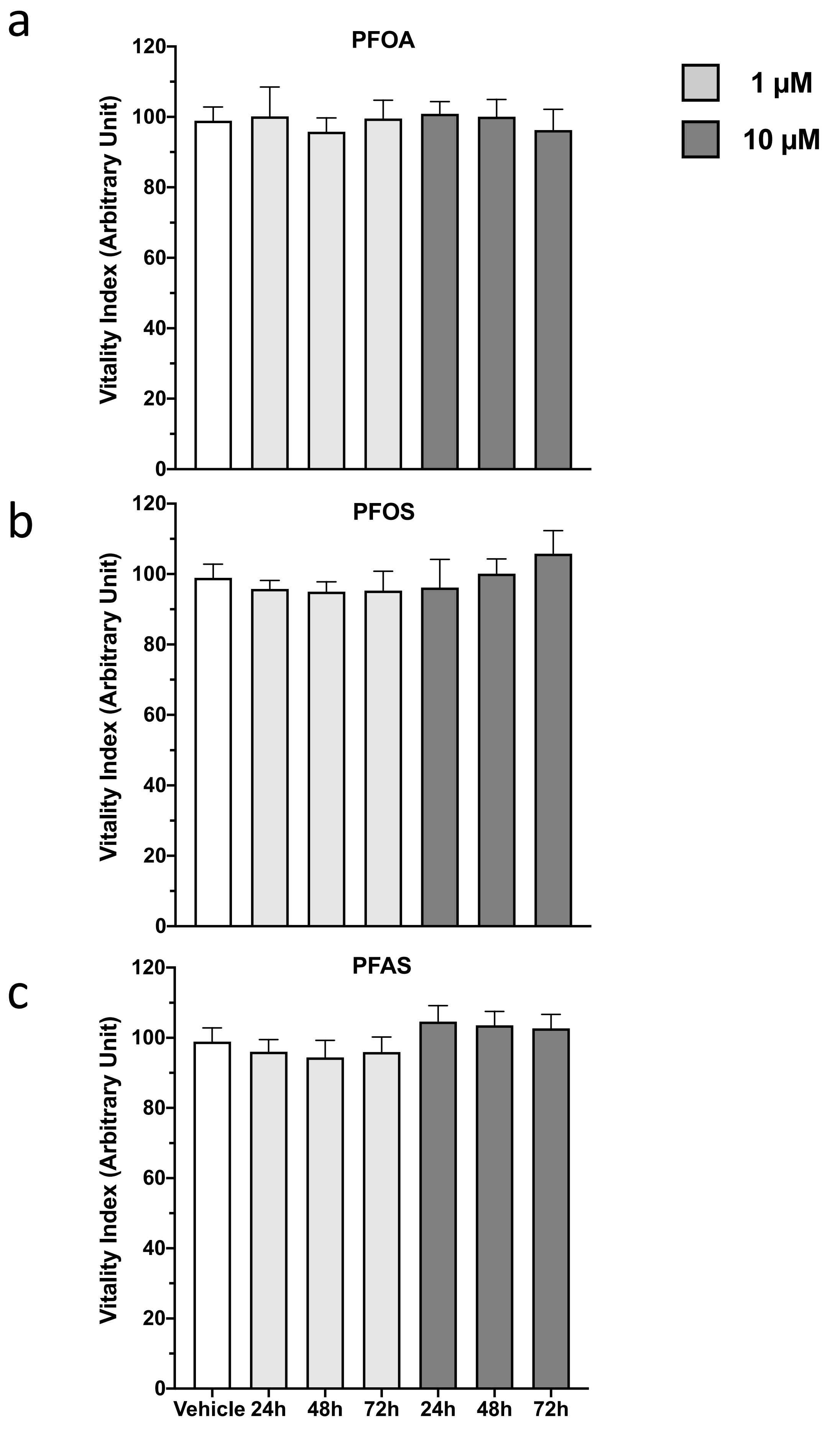

a
PFOA
b
PFOS
c
PFAS
Vitality Index (Arbitrary Unit)
0
20
40
60
80
100
120
Vehicle
24h
48h
72h
24h
48h
72h
1 μM
10 μM

Supplemental Figure S1. Adrenocortical cells viability is not affected by perfluoroalkyl substances.

HAC15 cell viability was evaluated by MTT assay at 3 different time points (24, 48 or 72 hours) after treatment with two different concentration, 1 μM (in light grey) or 10 μM (in dark grey), of PFOA (a), PFOS (b) and PFAS (PFOA + PFOS, c). Cell viability was >95% in all the conditions analysed. Data are reported as a percentage of the treated cells on the untreated controls. One-way ANOVA with Dunnett post hoc test was performed and no significant statistical difference was observed.
